# Development of an Optimized CXCR4-Targeting Theranostic Pair

**DOI:** 10.2967/jnumed.125.269933

**Published:** 2026-05

**Authors:** Daniel Kwon, Ingrid Bloise, Zhengxing Zhang, Nadine Colpo, Ryan Wilson, Ruiyan Tan, Helen Merkens, Jutta Zeisler, Joseph Lau, Kuo-Shyan Lin, François Bénard

**Affiliations:** 1 BC Cancer Research Institute, Vancouver, British Columbia, Canada; and; 2 Department of Radiology, University of British Columbia, Vancouver, British Columbia, Canada

**Keywords:** CXCR4, chemokine receptors, PET, SPECT, radiotheranostics

## Abstract

We developed a new C-X-C chemokine receptor 4 (CXCR4)–targeting radiolabeled peptide, [^68^Ga]Ga/[^177^Lu]Lu-BL34, using a novel and potent cyclic peptide based on structure–activity relationship studies of LY2510924. **Methods:** Candidate inhibitors were designed on the basis of structure–activity studies and synthesized using solid-phase techniques, and their CXCR4 binding was assessed using a cell-based assay. An optimized cyclic peptide sequence was modified with a Lys-cysteic acid linker and DOTA chelator to make BL34. Other analogs possessing ornithine or diaminopimelic acid linkers were also synthesized and assessed. All radiotracers were assessed in mice engrafted with a mantle cell lymphoma model (Z138) via PET/SPECT imaging and biodistribution studies. Therapeutic efficacy of [^177^Lu]Lu-BL34 was assessed in Z138-engrafted mice with groups of 30 and 60 MBq and a control. **Results:** The optimized cyclic peptide showed a 3-fold improvement in CXCR4 binding compared with that of LY2510924. [^68^Ga]Ga-BL34 showed high imaging contrast for the tumor at 1 and 2 h after injection. Biodistribution studies confirmed these results, with an uptake of 15.1 ± 3.1 %ID/g in the tumor at 1 h after injection and primarily renal excretion with a kidney uptake of 2.4 ± 0.4 %ID/g. SPECT imaging of [^177^Lu]Lu-BL34 showed similar results, with rapid renal excretion of [^177^Lu]Lu-BL34 from nontarget organs and relatively high uptake in tumors up to 72 h after injection. Biodistribution studies confirmed high tumor uptake at all time points, with low uptake across all nontarget organs. Blocking studies with LY2510924 further confirmed specificity. Therapy studies showed dose-dependent survival benefit with [^177^Lu]Lu-BL34 treatment, with metastatic recurrence in the treatment groups. Changing the side chain length at the linker attachment site did not affect the biodistribution or tumor uptake. **Conclusion:** We report a new CXCR4-targeting pharmacophore that can be used as a radiotheranostic. [^68^Ga]Ga-BL34 and [^177^Lu]Lu-BL34 showed excellent imaging and therapeutic properties in preclinical studies and are promising candidates for clinical translation.

## Visual Abstract

**Figure:**
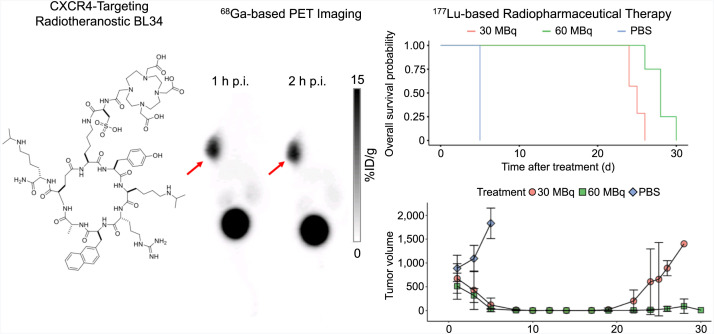

The C-X-C chemokine receptor 4 (CXCR4) is a G-protein-coupled receptor expressed in hematopoietic and immune cells to mediate cell trafficking (1–3). Its ligand is the stromal cell–derived factor-1 (SDF-1), expressed by organs to effect hematopoietic stem cell or immune cell migration (4). CXCR4 is expressed in multiple cancers (5). Paracrine signaling of SDF-1 contributes to the creation of a microenvironment conducive to cancer cell survival, migration, and invasion (1,6,7). SDF-1–expressing organs enable the successful seeding and growth of metastatic cells, contributing to poor outcomes (8,9). CXCR4-targeting pharmaceuticals are undergoing clinical trials for PET imaging and radiopharmaceutical therapy (RPT) (10,11).

The [^68^Ga]Ga-pentixafor/[^177^Lu]Lu- or [^90^Y]Y-pentixather pair have been evaluated in clinical studies (12–16). The pair showed uptake in multiple CXCR4-expressing pathologies (17). The objective of this study was to develop CXCR4-targeting radiotheranostics with improved tumor uptake and pharmacokinetics for PET imaging and RPT.

We reported CXCR4-targeting radiotracers derived from LY2510924 (**1**), a high-affinity and specific CXCR4 inhibitor (18). The first-generation [^68^Ga]Ga/[^177^Lu]Lu-BL01 showed high uptake in Burkitt lymphoma xenograft models with some accumulation in nontarget organ uptake (19). Subsequent linker structure–activity relationship studies showed that [^68^Ga]Ga/[^177^Lu]Lu-BL02 (**2**) had lower uptake in nontarget organs. This compound showed good therapeutic efficacy in a mantle cell lymphoma xenograft model (20–22).

To optimize a candidate for clinical translation, we sought to develop a peptide with improved pharmacologic inhibition of CXCR4 over LY2510924, which failed in phase 2 clinical trials (23,24). We hypothesized that modifying the peptide backbone position could result in improved tumor uptake and retention.

## MATERIALS AND METHODS

### Chemical and Radiochemical Synthesis

LY2510924 was purchased from MedChem Express. Details on the synthesis and labeling of the compounds can be found in the supplemental materials (available at http://jnm.snmjournals.org).

### In Vitro Competition Binding Assay

Details of the method used for measurement of half-maximal inhibitory concentration (IC_50_) values of compounds can be found in the supplemental materials.

### Animal Models

Animal experiments were performed in accordance with guidelines established by the Canadian Council on Animal Care, under a research protocol approved by the Animal Ethics Committee of the University of British Columbia. The Z138 mantle cell lymphoma cell line was used for in vivo studies using male NOD.Cg-Rag1*^tm1Mom^*Il2rg*^tm1Wjl^*/SzJ (NRG) mice. Further details are provided in the supplemental materials.

### PET/CT and SPECT/CT Imaging

PET/CT scans were performed on a Siemens Inveon, whereas SPECT/CT images were obtained using a MILabs scanner. The imaging procedures are described in the supplemental materials.

### Biodistribution

Under brief isoflurane sedation (2%–2.5% isoflurane in 2 L/min O_2_), the mice were injected intravenously with 0.8–3.0 MBq of ^68^Ga- or ^177^Lu-labeled peptides, allowed to roam freely afterward in their cage, and euthanized at the selected time points. Additional groups of mice received 7.5 µg (0.25–0.3 mg/kg) of LY2510924 intraperitoneally as a blocking control 15 min before radiotracer injection and were euthanized 1 h after injection.

### Therapy Studies

When the Z138 xenografts had grown to a volume of 600 ± 200 mm^3^, the mice were randomized into 3 groups (*n* = 7–8 each). Z138 xenograft mice were briefly sedated (2%–2.5% isoflurane in 2 L/min O_2_) and injected with either 30 or 60 MBq of ^177^Lu-labeled peptide or phosphate-buffered saline (100 µL). All groups were monitored for tumor volume, body weight, and behavior every other day for 60 d or until mice reached the volume endpoint (>1,500 mm^3^), loss of body weight (>15%), or behavioral signs of distress (e.g., lethargy, loss of appetite). Tumor sizes were measured using a 3-dimensional optical tumor scanner (TumorImager2; Biopticon).

### Statistical Analysis

Statistical analyses were performed by GraphPad Prism 8 and R version 4.2.2 (The R Foundation for Statistical Computing). The ROUT method was used to identify outliers (α = 0.01) (25). A 1-way ANOVA test, with a post hoc Tukey test, was used to check for differences between ^68^Ga-labeled peptides. A Welch *t* test was used to compare tumor and organ uptake values between the tumor values of ^68^Ga- and ^177^Lu-labeled peptides, and the tumor and organ uptake values of the ^177^Lu-labeled peptide were compared to those of [^177^Lu]Lu-BL02 ([^177^Lu]Lu-**2**) (21). Mixed modeling of log-transformed tumor volumes was used to determine the treatment effect of the ^177^Lu-labeled peptide in the therapy study. Reported *P* values were adjusted for multiple comparisons.

## RESULTS

**FIGURE 1.**
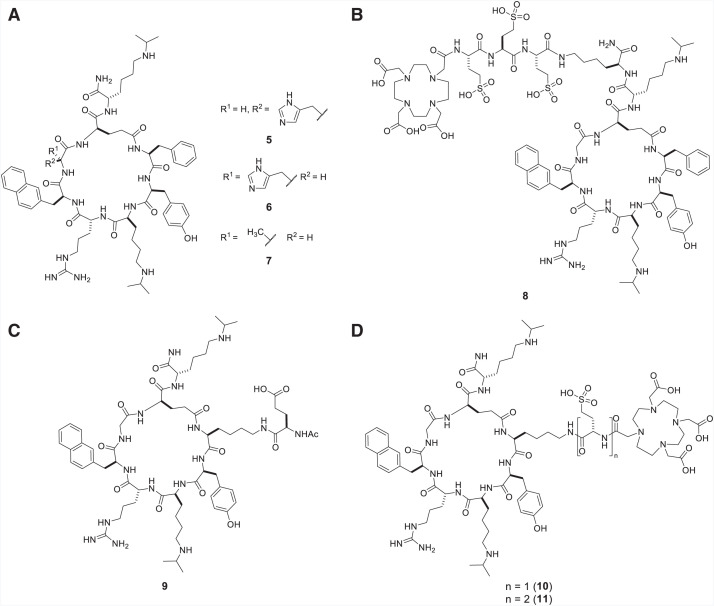
(A) Structures of novel cyclic peptides based on substitution of the Gly^2^ with His^2^ (**5**), D-His^2^ (**6**), and D-Ala (**7**). (B) Radiotheranostic candidate **8** using **7** as pharmacophore with decreased affinity for CXCR4. (C) Modified cyclic peptide **9** replacing Phe^7^ with Lys^7^(D-Glu-Ac) to evaluate potential sites of linker–chelator conjugation. (D) Two radiotheranostic candidates **10** and **11**, of which **10** (BL34) retained high affinity for CXCR4.

We reviewed other CXCR4-targeting inhibitors to inform potential modifications of LY2510924 (**1**) that may enhance binding affinity (Supplemental Fig. 1). We considered the cyclic peptide **3** (FC131), which serves as the targeting pharmacophore for pentixafor/pentixather (Fig. 1). However, **3** possesses similar amino acids in its sequence that are essential for binding (D-Tyr, Arg, and Nal) and nonessential Arg and Gly, all of which are present on LY2510924 (26). Recently, a series of cyclic pentapeptides was developed from a tripeptide motif found in SDF-1 and vMIP-II, a chemokine secreted by Kaposi sarcoma–associated herpesvirus; Ac-Arg-Ala-[D-Cys-Arg-2Nal-His-Pen]-COOH (**4**) showed high affinity for CXCR4 (Fig. 1) (27). Comparison of **4** and **1** revealed structural similarities, with the Arg-2Nal mimicking the D-Arg^4^-2Nal^3^ in **1** and the cross-linking motif (Pen-D-Cys) in a similar location compared with the D-Glu^1^-Phe^7^ lactam cross-link in **1** (Supplemental Fig. 1). A histidine residue present on **4** was chosen to be the starting point for our structure–activity relationship study and substituted over the Gly^2^ residue of **1**.

Although **5**, bearing a L-His^2^ in place of Gly^2^, showed no improvement in its affinity for CXCR4 (IC_50_ = 37.3 nM); **6**, with a D-His^2^, showed a 2-fold relative improvement in affinity for CXCR4 (IC_50_ = 10.1 nM) compared with LY2510924 (**1**) (IC_50_ = 20 nM) (Fig. 1A). We also synthesized **7**, with a D-Ala^2^ in place of D-His^2^, as a control compound to assess the contribution of the side chain to binding (Fig. 1A). Compound **7** possessed an IC_50_ of 6.7 nM, representing an approximate 3-fold improvement over **1**. As such, **7** was selected as our CXCR4-targeting pharmacophore for a new CXCR4-targeting radioligand.

Previously, we attached a lysine-based linker at the C-terminus of **1** to synthesize **2** (BL02) (21). A trianionic cysteic acid–based linker reduced kidney uptake (22). Using this strategy, we synthesized **8**, which had a significant decrease in CXCR4 affinity when conjugated to gallium (IC_50_ = 298 nM) (Fig. 1B). We looked at an alternate site on **7** for linker placement. We hypothesized that the Phe^7^ residue may be an appropriate site for linker conjugation. To test this hypothesis, we synthesized **9**, with the Phe^7^ switched out in place of Lys^7^(D-Glu-Ac). The Lys would act as an anchor for linker design and the N-terminus was conjugated to a D-Glu-Ac to mimic the anionic linker moiety needed to modulate the pharmacokinetics of the desired radiotracer and the amide bond necessary for conjugation. **9** showed a binding potency similar to that of **7** (IC_50_ = 7.4 nM; Fig. 1C). On the basis of this result, we synthesized **10** (hereafter referred to as BL34) and **11**, with 1 and 2 cysteic acids, respectively, conjugated to the ϵ-*N*-amine of Lys. Both compounds had a DOTA group to enable labeling with ^68^Ga and ^177^Lu (Fig. 1D). Although Ga-**11** showed good binding (IC_50_ = 24.2 ± 3.5 nM), Ga-BL34 showed better affinity for CXCR4 (IC_50_ = 10.3 ± 5.7 nM). Lu-BL34 retained good binding to CXCR4 (IC_50_ = 24 ± 11 nM). Neither compound bound significantly to mouse CXCR4. Ga-BL34 and Lu-BL34 showed no measurable inhibition constants or poor binding in the high micromolar to millimolar range (IC_50_ = 0.28 ± 0.4 mM and 3.4 ± 6.9 mM, respectively).

**FIGURE 2.**
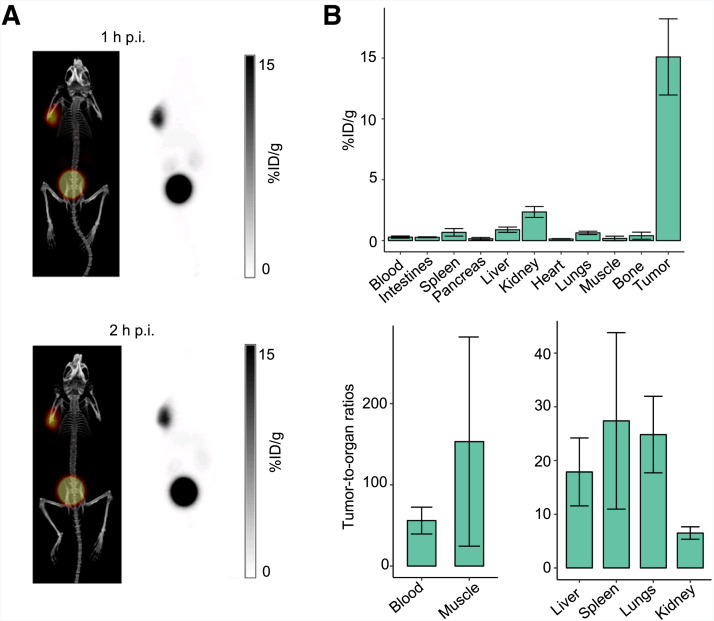
(A) Maximum-intensity projection PET/CT and PET images of Z138-bearing NRG male mouse injected with [^68^Ga]Ga-BL34 and imaged longitudinally at 1 and 2 h after injection. (B) Ex vivo biodistribution studies at 1 h after injection of [^68^Ga]Ga-BL34 in Z138-bearing NRG male mice. p.i. = postinjection.

With the development of **10** (BL34), we conducted radiolabeling experiments with ^68^Ga for PET imaging. [^68^Ga]Ga-BL34 (*n* = 7) was obtained at high molar activity (346 ± 206 GBq/µmol), radiochemical yield (73.5 ± 1.9%), and radiochemical purity (>99%). We evaluated [^68^Ga]Ga-BL34 in Z138 mantle cell lymphoma xenograft–bearing NRG male mice (21). PET imaging of mice injected with [^68^Ga]Ga-BL34 showed high uptake in the tumor at 1 h (SUV_max_, 5.0) and 2 h after injection (SUV_max_, 4.9) (Fig. 2A). There was low uptake elsewhere, notably in the blood pool of the heart at 1 h (SUV_max_, 0.19) and 2 h after injection (SUV_max_, 0.079) and in the liver at 1 h (SUV_max_, 0.28) and 2 h after injection (SUV_max_, 0.23). Excretion was primarily renal, with the kidney having the highest uptake of the nontarget organs at 1 h (SUV_max_, 0.91 and 0.81 for left kidney and right kidney, respectively) and 2 h after injection (SUV_max_, 0.38 and 0.43 for left kidney and right kidney, respectively), with high accumulation in the bladder.

Ex vivo biodistribution studies of [^68^Ga]Ga-BL34 showed high uptake at 1 h after injection in the tumor (15.1 ± 3.1 %ID/g) (Fig. 2B; Supplemental Table 1). Excretion was primarily renal, as the kidneys showed the highest uptake (2.4 ± 0.5 %ID/g) of all nontarget organs; there was minimal uptake in the liver and bowel (liver, 0.90 ± 0.22 %ID/g; small intestines, 0.27 ± 0.04 %ID/g). Accordingly, tumor-to-organ ratios were high (tumor-to-blood ratio, 56.0 ± 16.5; tumor-to-muscle ratio, 153 ± 129; tumor-to-kidney ratio, 6.5 ± 1.2). At 2 h after injection, there was good retention of [^68^Ga]Ga-BL34 in the tumor and rapid clearance from the rest of the nontarget organs (Supplemental Table 1).

**FIGURE 3.**
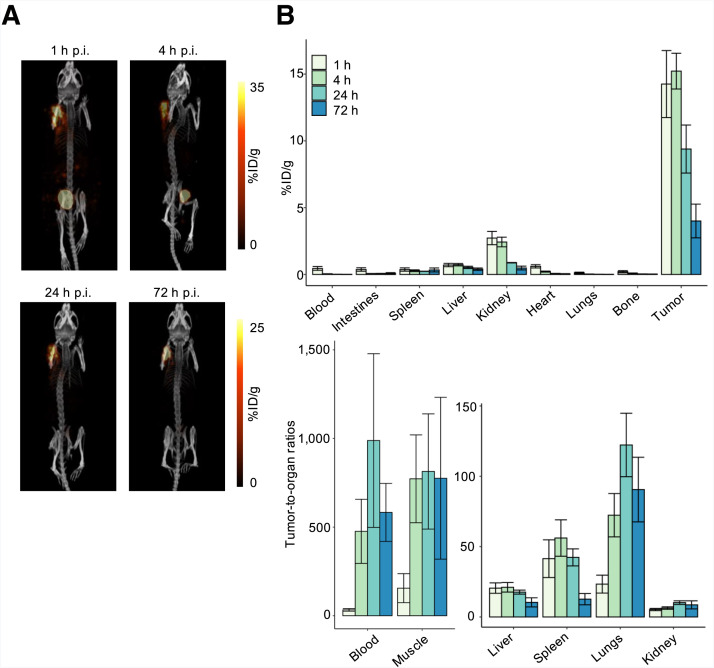
(A) Maximum-intensity projection SPECT/CT images of Z138-bearing NRG male mouse injected with [^177^Lu]Lu-BL34 and imaged longitudinally at 1, 4, 24, and 72 h after injection. (B) Ex vivo biodistribution studies at 1, 4, 24, and 72 h after injection of [^177^Lu]Lu-BL34 in Z138-bearing NRG male mice. p.i. = postinjection.

We evaluated BL34 for therapy. [^177^Lu]Lu-BL34 (*n* = 2) was synthesized and purified at a high molar activity (249 ± 65 GBq/µmol), radiochemical yield (41.8 ± 22.8%), and radiochemical purity (>95%). SPECT imaging of Z138-bearing xenograft mice injected with [^177^Lu]Lu-BL34 showed high uptake in the tumor, with low uptake in other organs (Fig. 3A). [^177^Lu]Lu-BL34 was excreted by the kidneys, with accumulation in the bladder. At later time points, the kidneys were no longer visualized, with decreasing activity in the bladder. The tumor was still clearly visualized at 72 h. Biodistribution studies confirmed these results (Fig. 3B; Supplemental Table 2). [^177^Lu]Lu-BL34 had high uptake in the tumor, peaking at 4 h after injection, with subsequent washout of the radiotracer at 24 and 72 h after injection (14.3 ± 2.5 %ID/g at 1 h, 15.2 ± 1.3 %ID/g at 4 h, 9.4 ± 1.8 %ID/g at 24 h, and 4.0 ± 1.3 %ID/g at 72 h). There was higher tumor uptake at 24 and 72 h after injection in the SPECT images compared with the biodistribution study. This was due to volume loss of the xenograft, leading to an increase in radioactivity per volume of the tumor, secondary to the therapeutic effects of the higher administered dose of [^177^Lu]Lu-BL34 for imaging. Specificity was confirmed with preinjection of LY2510924, showing a 63% decrease in uptake in Z138 xenografts. There was rapid washout of the radiotracer from circulation with high tumor-to-blood ratios measured at each time point. The kidney activity peaked at 1 h (2.7 ± 0.5 %ID/g), decreasing at each time point to 0.48 ± 0.14 %ID/g at 72 h. The tumor-to-kidney ratio of [^177^Lu]Lu-BL34 was consistently high, peaking at 10.2 ± 1.3 at 24 h.

Compared with [^177^Lu]Lu-**2** (BL02) with a triglutamic acid linker (21), [^177^Lu]Lu-BL34 had similar tumor uptake and similar or lower uptake in other organs. This translated into statistically higher tumor-to-organ ratios such as bone (24 and 72 h after injection, *P* < 0.01), liver (24 h after injection, *P* < 0.01), and lungs (24 and 72 h after injection, *P* < 0.01).

**FIGURE 4.**
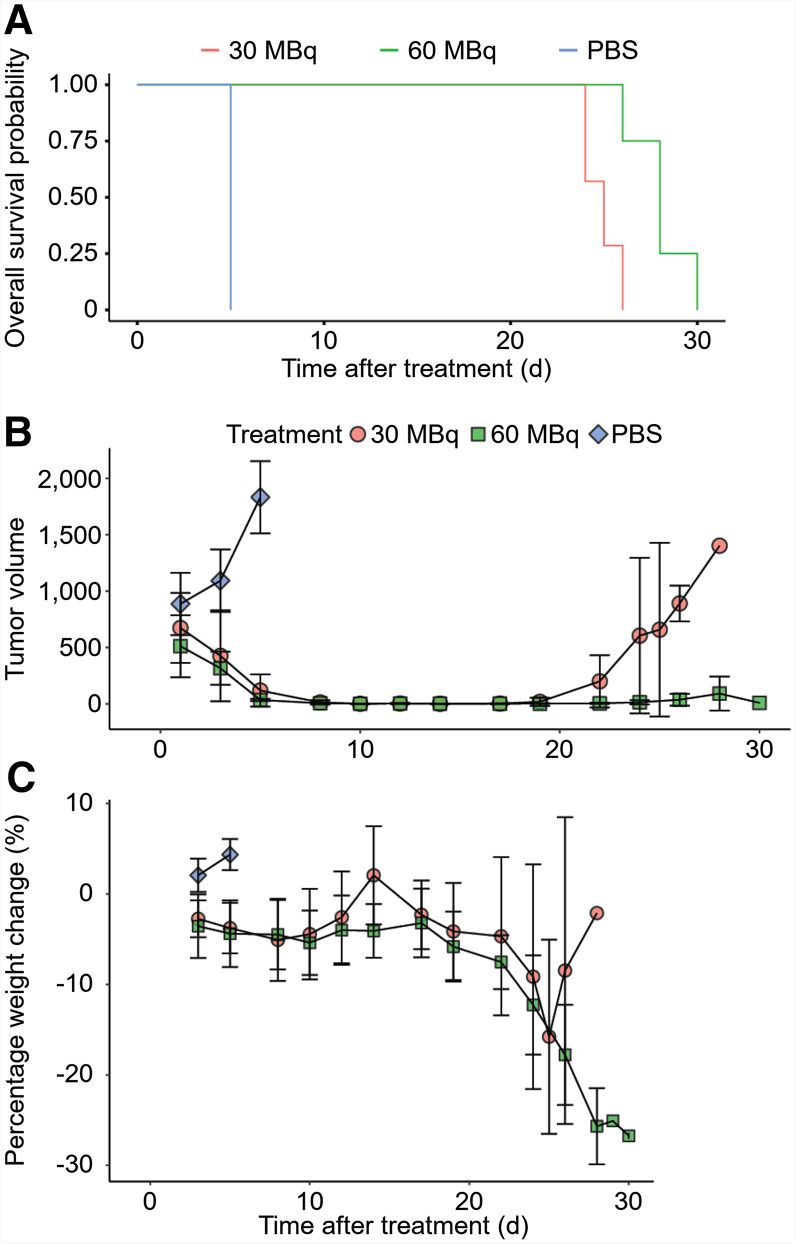
(A) Kaplan–Meier survival curve showing difference in survival between control and treatment groups (30 or 60 MBq of [^177^Lu]Lu-BL34). (B) Mean and SD of Z138 tumor volumes of mice in control and treatment groups. (C) Percentage change in weight of mice in control or treatment groups as function of time until termination. PBS = phosphate-buffered saline.

Mice bearing Z138 xenografts were administered 30 MBq or 60 MBq of [^177^Lu]Lu-BL34 or a phosphate-buffered saline control and were monitored longitudinally. A Kaplan–Meier survival curve analysis showed a significant difference in survival between all groups (*P* = 4.45 × 10^−7^) (Fig. 4A). The control group rapidly reached their endpoint after 5 d. Both treatment groups showed a decrease in tumor volume until complete regression after a median of 10 d (Fig. 4B). Mild weight loss was observed in these groups by 10 d (Fig. 4C). At 20 d, mice in the 30-MBq group showed local recurrence and were subsequently euthanized when tumors reached greater than 1,500 mm^3^. Mice in the 60-MBq group showed no or minimal tumor recurrence at the inoculation site but reached humane endpoints at 28–30 d due to behavioral changes or weight loss. Both groups showed progressive weight loss and behavioral findings, such as decreased food intake, lethargy, and loss of hindleg movement; the 30-MBq group was euthanized because of tumor volume, whereas the 60-MBq group was euthanized due to weight loss/behavioral changes. This behavioral change and weight loss were consistent with a previous study (21). Gross evaluation of organs showed several sites of metastasis such as the liver. The effects of metastatic involvement included findings such as dilatation of the small intestines, likely secondary to obstruction by metastatic deposits in the peritoneum (Supplemental Fig. 2).

To facilitate future studies, we assessed the in vivo effect of shortening the alkyl side chain of our Lys linker; this could enable the placement of an orthogonal linker to conjugate a second moiety for pharmacokinetic modulation. We therefore synthesized **12** and **13**, replacing the Lys^7^ with ornithine and diaminopropionic acid, respectively (Supplemental Fig. 3). Both compounds were labeled with [^68^Ga]GaCl_3_ at a radiochemical yield of 81.3% and 72.7%, respectively, and a radiochemical purity of greater than 99%.

**FIGURE 5.**
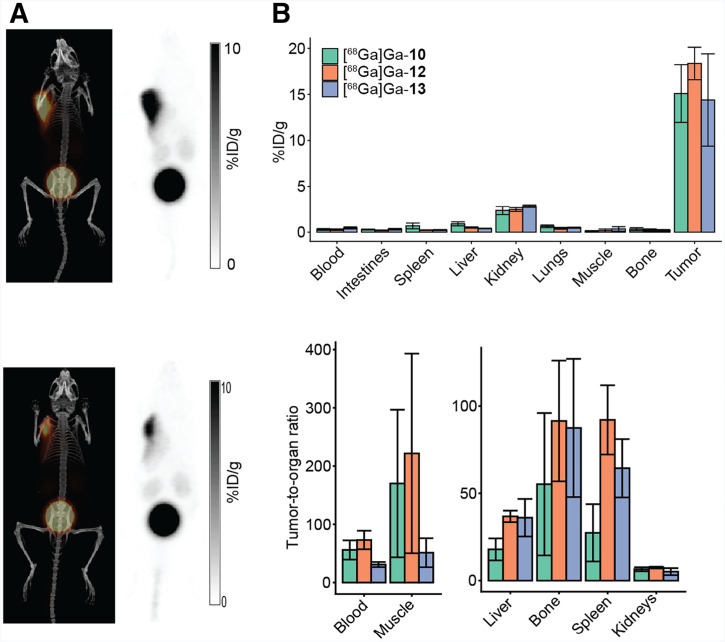
(A) Maximum-intensity projection PET/CT and PET images of Z138-bearing NRG male mice injected with either [^68^Ga]Ga-**12** (ornithine amino acid linker) or [^68^Ga]Ga-**13** (diaminopropionic acid linker). (B) Comparative ex vivo biodistribution study of [^68^Ga]Ga-**10**, [^68^Ga]Ga-**12**, and [^68^Ga]Ga-**13** in Z138-bearing NRG male mice.

We performed a small pilot study to ensure that the tumor-targeting capability and pharmacokinetics of BL34 were preserved in [^68^Ga]Ga-**12** and [^68^Ga]Ga-**13**. In vivo imaging and ex vivo biodistribution studies showed a performance similar to that of [^68^Ga]Ga-BL34. On PET imaging, [^68^Ga]Ga-**12** and [^68^Ga]Ga-**13** showed high signal in the tumor and in the bladder, indicating renal excretion, and little to no uptake throughout most nontarget organs (Fig. 5A). Ex vivo biodistribution studies confirmed these results (Fig. 5B; Supplemental Tables 3 and 4). [^68^Ga]Ga-**12** and [^68^Ga]Ga-**13** had a tumor uptake of 18.4 ± 1.8 %ID/g and 14.4 ± 5.0 %ID/g, respectively. No statistically significant difference in tumor uptake was found among the 3 tracers.

## DISCUSSION

We reported and characterized new CXCR4 radiotracers based on the development of an optimized cyclic peptide. BL34, along with derivatives substituting lysine for diaminopropionic acid and ornithine, had suitable pharmacokinetic properties to image and treat CXCR4-expressing cancers. We reported [^68^Ga]Ga/[^177^Lu]Lu-**2** (BL02) as a radiotheranostic pair for CXCR4-targeted PET imaging and RPT, which compared favorably with [^68^Ga]Ga-pentixafor, with higher tumor uptake and lower nontarget organ uptake. In the current study, we assessed whether modifications to the pharmacophore could further enhance its in vivo performance. This resulted in a cyclic peptide with improved affinity for CXCR4.

The improved binding affinity by modifying Gly^2^ to D-Ala^2^ is likely due to the restriction of the cyclic peptide conformation. This would decrease the entropic penalty of having to first adopt the conformation before binding to CXCR4, thereby enhancing its overall binding affinity for the target. This is best exemplified in work by Chen et al., wherein replacing a glycine residue of a bicyclic peptide into several D-amino acids resulted in increased affinity and in vitro stability (28).

Unlike **2**, peptide **7**, with the Gly^2^ to D-Ala^2^ modification from the original peptide, could not accommodate a lysine residue at its C-terminus. This hints at subtle differences in binding conformation between the 2 peptides. Modification of the Phe^7^ to bear a lysine instead with the requisite amine group for conjugation was noninferior; further placement of the linker and metal–chelator complex resulted in a CXCR4-targeting radiotheranostic with excellent in vivo capabilities. We opted for cysteic acid as previous work showed that replacement of the triglutamate linker with a tricysteic acid linker led to lower uptake in the kidneys (22). This likely explains the lower kidney uptake in [^177^Lu]Lu-BL34 compared with [^177^Lu]Lu-**2** (BL02).

In vivo assessment of [^68^Ga]Ga/[^177^Lu]Lu-BL34 showed excellent uptake in tumors and low nontarget organ uptake, with rapid clearance. [^177^Lu]Lu-BL34 showed low nontarget organ uptake and rapid circulatory clearance. We further showed that [^177^Lu]Lu-BL34 was either noninferior or had potentially lower off-target organ uptake than [^177^Lu]Lu-**2** (BL02) (21).

It must be noted that LY2510924 has poor affinity for murine CXCR4 over human CXCR4 (29). Ga-BL34 and Lu-BL34 did not significantly bind to the murine CXCR4 receptor. As such, preclinical mouse models may not accurately reflect the human distribution of BL34 for imaging and therapy; however, this is an issue that extends to many CXCR4-targeting radiotracers. [^68^Ga]Ga-pentixafor and [^177^Lu]Lu-pentixather, clinically validated CXCR4-targeting tracers, also do not bind or have very poor binding to murine CXCR4 (30). On clinical translation, [^68^Ga]Ga-pentixafor showed no concerning uptake in CXCR4-expressing organs such as the liver or lungs (31). Nonetheless, true validation of our radiotheranostic’s potential for RPT will come from clinical trials conducted in humans.

[^68^Ga]Ga-pentixafor has lower uptake in many solid cancers compared with [^18^F]FDG, notably in breast cancer, head and neck squamous cell cancers, and melanomas (32–35). Our previous work showed that [^68^Ga]Ga-**2** (BL02) had high tumor uptake and tumor-to-organ ratios in vivo (21). The improved performance of BL34 over BL02 and the in vivo data in this study suggest a potential for improved radiotracer delivery to cancers with lower expression of CXCR4. Several other CXCR4 radioligands use small molecule–based or peptide-based pharmacophores; [^64^Cu]Cu-AMD3100 (NCT02069080) and [^18^F]AlF-NOTA-NFB (36) are 2 other CXCR4-targeting agents that have or are undergoing clinical evaluation. Both have potential for theranostic applications. Prospective clinical studies will be required to assess the clinical utility of the various CXCR4-targeting radioligands.

Given the high tumor-to-organ ratios of [^177^Lu]Lu-BL34, we assessed its efficacy in therapy studies. We showed in a previous study that CXCR4-targeted RPT induced regression in Z138 xenograft–bearing mice; however, the mice experienced both primary site recurrence and metastatic disease (21). We therefore assessed whether higher doses could prevent recurrence. As expected, [^177^Lu]Lu-BL34 was highly efficacious in causing tumor regression compared with the control group. Patients with mantle cell lymphoma generally have poor outcomes (21,37–39). Furthermore, lymphoma subtypes, such as double hit diffuse large B-cell lymphomas and Burkitt lymphomas, are reported to have elevated CXCR4 expression and can have very poor outcomes (40,41). Although xenograft models in immunocompromised mice poorly recapitulate the tumor biology within human malignancies, with respect to cell types (e.g., immune and stromal cell infiltration) and tumor characteristics (e.g., vasculature and hypoxia), this single-dose therapy study demonstrates a potential for clinical translation.

## CONCLUSION

We report a series of radiopharmaceuticals based on a novel high-affinity CXCR4-targeting peptide derived from the structure of LY2510924. Investigations of [^68^Ga]Ga/[^177^Lu]Lu-BL34 showed excellent uptake in CXCR4-expressing xenografts with low nontarget organ uptake. In vivo imaging and RPT studies demonstrated [^68^Ga]Ga/[^177^Lu]Lu-BL34’s potential to image patients with CXCR4-expressing cancers, identify suitable patients for CXCR4-targeted therapy, and treat them with RPT. Thus, [^68^Ga]Ga/[^177^Lu]Lu-BL34 has significant clinical translational potential to assess patients with CXCR4-expressing malignancies.

## DISCLOSURE

This study was supported by the Canadian Institutes of Health Research (FDN-148465) and the BC Cancer Foundation. Daniel Kwon is supported by a CIHR Doctoral Research Award, the Laurel L. Watters Research Fellowship, and the IODE War Memorial Scholarship for Doctoral Study. Daniel Kwon, Zhengxing Zhang, Kuo-Shyan Lin, and François Bénard are coinventors of a filed patent (WO2020210919A1) that includes some of the disclosed work in this article and has been licensed to Alpha-9 Oncology. Daniel Kwon received consulting fees from Alpha-9 Oncology. Kuo-Shyan Lin, François Bénard, and Zhengxing Zhang are shareholders of Alpha-9 Oncology. Kuo-Shyan Lin and François Bénard received consulting fees and research funding from Alpha-9 Oncology and are cofounders of the company. No other potential conflict of interest relevant to this article was reported.

## Notes

KEY POINTS **QUESTION:** Can an existing CXCR4-targeting pharmacophore be improved with respect to CXCR4 affinity to develop radiotheranostic agents with high tumor uptake and low nontarget organ uptake sufficient for clinical translation? **PERTINENT FINDINGS:** A targeted modification of Gly to D-Ala on LY2510924 improved the affinity for CXCR4 3-fold. Based on this, the new pharmacophore, [^68^Ga]Ga/[^177^Lu]Lu-BL34, was developed and showed excellent uptake in CXCR4-expressing tumors with low nontarget organ uptake, with the former showing therapeutic efficacy in a therapeutic study in xenograft mice. **IMPLICATIONS FOR PATIENT CARE:** The excellent in vivo performance of [^68^Ga]Ga/[^177^Lu]Lu-BL34, based on a new cyclic peptidic pharmacophore, highlights its potential for clinical translation for sensitive detection and RPT of CXCR4-expressing cancers.
